# Neoantigens and the tumor microenvironment play important roles in the prognosis of high-grade serous ovarian cancer

**DOI:** 10.1186/s13048-022-00955-9

**Published:** 2022-01-29

**Authors:** Hua Yang, Mei Yu, Sen Zhong, Yan You, Fengzhi Feng

**Affiliations:** 1 Department of Obstetrics and Gynecology, National Clinical Research Center for Obstetric & Gynecologic Diseases, Peking Union Medical College Hospital, Chinese Academy of Medical Sciences & Peking Union Medical College, No.1 Shuai Fu Yuan, Wang Fu Jing Street, Beijing, 100730 China; 2 Department of Pathology, Peking Union Medical College Hospital, Chinese Academy of Medical Sciences & Peking Union Medical College, No.1 Shuai Fu Yuan, Wang Fu Jing Street, Beijing, 100730 China

**Keywords:** Neoantigen, High-grade serous ovarian carcinoma, Immune microenvironment

## Abstract

**Background:**

Despite the consistency of patient age, disease stage and treatment options, the prognosis of different high-grade serous ovarian carcinoma (HGSOC) patients is different. Here, we sought to measure predictive biomarkers for distinct responses to platinum-based chemotherapy and immunotherapy benefits.

**Methods:**

Sixteen HGSOC patients receiving debulking surgery and adjuvant first-line combination chemotherapy at Peking Union Medical College Hospital (PUMCH) were enrolled. Whole exome sequencing (WES) and RNA-seq were performed on tumor and normal tissues of these patients.

**Results:**

The tumor mutational burden (TMB) and intratumoral heterogeneity (ITH) of the platinum-resistant group were significantly higher than those of the platinum-sensitive group (*P*=0.0321 and *P*=0.0452, respectively). TMB, neoantigen and ITH had certain predictive value according to the area under the receiver operating characteristic (ROC) curve (AUC 0.7778 for TMB, 0.7619 for neoantigen, 0.7778 for ITH). The infiltration of other immune cells in tumor tissues was different between the two groups, but the difference was not significant. Univariate Cox proportional hazard analysis revealed poorer progression-free survival (PFS) for those patients who carried a higher number of neoantigens (*P* =0.0069), higher TMB (*P* =0.0083), and higher ITH (*P* =0.0249). Further Kyoto Encyclopaedia of Genes and Genomes (KEGG) analysis indicated the Differentially expressed genes (DEGs) in platinum-resistant and platinum-sensitive patients were mainly enriched in the phosphatidylinositol 3-kinase-Akt (PI3K-Akt) signaling pathway and focal adhesion pathway, which are associated with platinum resistance.

**Conclusions:**

Higher TMB, neoantigen and ITH may account for the worse prognosis of patients with platinum-based chemotherapy and higher TMB was observed in the platinum-resistant group, which could make the patients in the platinum-resistant group to be the better candidates for immunotherapy.

**Supplementary Information:**

The online version contains supplementary material available at 10.1186/s13048-022-00955-9.

## Introduction

Epithelial ovarian cancer (EOC) is the most lethal gynecological cancer, for which the standard treatment is cytoreductive surgery followed by platinum-taxane combination chemotherapy. After therapy, the overall five-year survival rate has been only 28-46% for the last 30 years, and one of the main factors affecting survival is the response to platinum-based chemotherapy [1]. It has been reported that platinum-resistant patients account for 25% of cases and have a very low five-year survival rate.

High-grade serous carcinoma (HGSC) is the most common histological subtype of ovarian cancer and is highly aggressive and grows rapidly. Sensitivity to first-line chemotherapy is associated with the prognosis of patients. However, different responses to platinum-based chemotherapy are observed even among patients with similar clinical characteristics and disease stages. Similar to other cancers, the presence of intratumoral heterogeneity (ITH) and mutation is likely associated with the responsiveness of HGSC to platinum-based chemotherapy, which has inspired efforts to explore predictive biomarkers for the response rate to platinum-based chemotherapy.

Genomic predisposition to EOC shows that HGSC is chromosomally highly unstable and harbors tumor protein 53 (TP53) mutations and mutations of breast cancer 1 (BRCA1) and breast cancer 2 (BRCA2) [2]. Although expression of the BRCA gene is a predictive biomarker for outcome on poly ADP-ribose polymerase (PARP) inhibitors, it is not a biomarker for the response rate to platinum-based chemotherapy. Therefore, other biomarkers that can be better predict beneficial outcomes of chemotherapy are needed. Until now, in addition to BRCA, only homologous recombination deficiency (HRD) has been approved as a predictive biomarker by the Food and Drug Administration (FDA). In recent years, with the development of targeted therapy and immunotherapy, it has been reported that the discovery of neoantigen biomarkers and the application of immunotherapy can greatly improve the prognosis of pancreatic cancer, melanoma, rectal cancer and lung cancer. However, it remains unclear whether neoantigens can be used as a predictive biomarkers for the response rate to platinum-based chemotherapy or immunotherapy benefits in HGSC patients.

To explore these issues, platinum-resistant and platinum-sensitive patients with similar clinical characteristics and the same treatment were collected retrospectively and studied. Additionally, we aimed to try to preliminarily explore which patients could benefit from immunotherapy. Furthermore, we analyzed bioinformation of HGSC patients from The Cancer Genome Atlas (TCGA) database, to further verify the significance of this study in a larger number of HGSC patients.

## Materials and methods

### Study population

We retrospectively reviewed data of patients with EOC receiving initial treatment with cytoreductive surgery and adjuvant first-line combination chemotherapy at Peking Union Medical College Hospital (PUMCH) between March 1, 2015 and December 31, 2017. Patients eligible for inclusion in this study were those with: (1) HGSC, (2) optimal primary debulking surgery (PDS); (3) adjuvant chemotherapy of the TC regimen with carboplatin (area-under-the-curve, AUC 5) and paclitaxel (175 mg/m2 intravenously over 3 h) every 21 days for 6 cycles; and (4) regular follow-up after completing treatment. Women who received suboptimal cytoreductive surgery or non-TC regimen chemotherapy were excluded. In addition, women with comorbidities, such as diabetes mellitus, hypertension, and heart disease, were also excluded due to poor tolerance to standard combination chemotherapy.

Approval from the Institutional Review Board (JS-1936) at PUMCH was obtained. The study time period ended on December 31, 2017 to ensure at least 2 years of posttreatment follow-up for all patients at the time of chart review.

To define predictive biomarkers for the response rate to platinum-based chemotherapy, we collected matched cohorts of patients with stage III-IV HGSC who were defined as platinum-sensitive (disease recurrence was 12 months or longer after completing their last first-line platinum-based chemotherapy, *n* =11) with longer survival versus platinum-resistant (the disease recurrence or refractory was within 6 months after completing of their last first-line platinum-based chemotherapy, *n* = 11). Prior to generating the matched cohorts, women with unavailable relapse time or relapse at 6-12 months were excluded. The clinical characteristics of cohorts were matched using age, stage, grade, amount of residual disease, chemotherapy exposure and comorbidity score.

### Neoantigen predictions analysis

To determine the neoantigen frequency in HGSCs, WES was performed on embedded tumor and matched normal tissues. Tumor and matched normal samples were collected at the time of surgery.

Tumor and matched normal DNA were extracted using the GeneRead DNA FFPE Kit from formalin-fixed paraffin-embedded (FFPE) tissues. Libraries were constructed by the Agilent SureSelect ^XT^ Human All Exon V6 (Agilent Technologies) and sequenced with next-generation sequencing. Genomic DNA was fragmented, end-repaired, adenylated at the 3’ ends, end-connected, amplified, purified, size-selected in the process of library construction, and then sequenced on the Illumina X10 platform (Illumina Inc., San Diego, CA, USA). WES data underwent mutation analysis and human genome build hg19 was used as the reference genome. Somatic SNVs and InDels were analyzed via GATK MuTect2 (version 4.1, default parameters). The sequenced reads were realigned to the hg19 by Burrows-Wheeler Aligner BWA (version 0.7.15, default parameters, BWA-MEM algorithm) to enhance the validity of SNVs.

Tumor RNA was extracted from FFPE tissues using the RNeasy FFPE Kit (Qiagen). Libraries were constructed using a TruSeq RNA Exome kit and sequenced with NGS. Total RNA was fragmented, reverse transcribed into complementary DNA, base ‘A’ was added in the 3′ ends, adapter connected, amplified and purified, and then sequenced on the Illumina X10 platform (Illumina Inc., San Diego, CA, USA) to generate 2×150bp paired-end reads. Raw data were filtered by removing sequencing reads containing adaptor sequences and low-quality reads, which have too many Ns (>5%) and low-quality bases (>15% bases with quality ≤19), to obtain the clean reads. Clean reads were mapped to the hg19 human genome using Bowtie2 (version 2.2.4) software from Tophat2 (version 2.0.10) with default parameters. The program Cufflinks (version 2.2.1, default parameters) was used to calculate the expression levels of genes in terms of reads per kilobase per million reads (FPKM).

Human leukocyte antigen (HLA) typing was acquired from normal DNA using OptiType (version 1.3.1, default parameters). The cancer immunotherapy pipeline pVACseq (version 4.0.10, default parameters) was used to identify neoantigens [3]. Peptide-MHC affinity for half maximal inhibitory concentration (IC_50_) values was predicted using NetMHC (version 2.22.1, default parameters) or PickPocket (version 2.22.1, default parameters). IC_50_<500 nM is considered as an accepted standard criterion in the field for predicting binders, and IC_50_≤150 nM is accepted as a strong binder [4]. Mutated peptides with a binding affinity of IC_50_<500 nM were regarded as candidate neoantigens, and mutated peptides (IC_50_≤150 nM) were regarded as strong-quality neoantigens [5–7]. Neoantigen expression was confirmed in any such neoantigen with RNA-seq counts ≥1 [7, 8]. This study aimed to identify strong-quality neoantigens (mutated peptides of IC_50_≤500 nM).

### Data acquisition and processing in the TCGA database

Somatic mutation data and clinical information of ovarian cancer patients from TCGA were downloaded from the GDC data portal (https://portal.gdc.cancer.gov/). Patients included in the analysis were required to meet all of the following criteria: (1) HGSC; (2) III~IV stage; (3) no macroscopic disease or tumor residual disease 1~10mm during PDS; (4) PFS<6 months or >12 months; and (5) postoperative adjuvant chemotherapy with the TC regimen. Overall, 93 patients were selected for analysis, including 13 patients with PFS <6 months (platinum-resistant group) and 80 patients with PFS >12 months (platinum-sensitive group). The TMB for each patient was estimated as the number of variants per exon length.

### Calculating the intratumoral heterogeneity value of each patient

Based on the MATH algorithm, the ITH value of each patient was evaluated [9]. In brief, each patient’s ITH value was calculated from the median absolute deviation (MAD) and the median of its mutant-allele fractions at tumor-specific mutated loci (ITH value=100*MAD/median).

### Assessment of immune infiltration

Based on the gene expression (FPKM), an R package—ESTIMATE (version 1.0.13, default parameters) was used to estimate the fraction of stromal and immune cells in tumor samples. ‘Immune score’ aimed to represent the infiltration of immune cells in tumor tissue [10]. Additionally, an MCP-counter method (version 1.1.0, default parameters) was used to evaluate the absolute intratumor cell abundance of 10 cell types (8 immune and 2 stromal cell populations) in each tumor tissue [11].

### Differentially expressed genes (DEGs) analysis and functional enrichment analysis

The program Cuffdiff (version 2.2.1, default parameters) was used to calculate the expression levels of genes in terms of FPKM and the P value for DEGs based on two-tailed unpaired Student’s t-test. Genes with fold change >2, *P*-value <0.05 were deemed significant DEGs. Then, KEGG functional enrichment analysis of the DEGs was performed with KOBAS (version 3.0, default parameters). The enrichment results with a *P* value <0.05 were considered statistically significant.

### Statistical analysis

Statistical analysis was performed using SPSS 21.0 software (IBM Corporation, New York). Parametric data were calculated by t test or Fisher’s exact test. All correlation analyses were performed with Pearson’s correlation coefficient. In all analyses, a two-sided *P* value<0.05 was considered statistically significant. Figures were drawn using GraphPad Prism 7.0 software (San Diego, USA) or R (https://cran.r-project.org/).

## Results

### Patient characteristics and mutation identification

In total, 22 patients were collected, and 16 patients were ultimately analyzed because six patients’ WES or RNA data could not be used, including 4 in the platinum-resistant group and 2 in the platinum-sensitive group. The characteristics of the16 patients are shown in Table 1. Clinical data were matched well and not significantly different between platinum-resistant and platinum-sensitive groups. The median age of the platinum-resistant (R) group was 60±7.4 years old, and that of platinum-sensitive (S) group was 52±11.1. There was no significant difference between the two groups (*P*=0.115). The mean value of preoperative serum CA125 for patients in the S group was 1086.9±599.9 IU/ml, while the mean value in Group R was 1586.2±1489.7 IU/ml, with no significant difference between the two groups (*P*=0.380). The clinical stages of the two groups were analysed and there was no significant difference between the two groups (*P*=0.774). Meanwhile, the degree of surgical excision also showed no difference between the two groups (*P*=0.898). The progression-free survival (PFS) of Group R ranges from 0 to 5 months, and the PFS of Group S ranged from 13 to 22 months. In addition, the mutational spectrum of these 16 patients is characterized in Supplementary Fig. S1. Only the top14 frequently mutated genes (mutated in at least 3 patients) are shown, including *MUC4* (56.25%, 9/16), *MUC17* (50.00%, 8/16), *TP53* (31.25%, 5/16), *HRNR* (31.25%, 5/16), *FLG* (31.25%, 5/16), *ZNF253* (25.00%, 4/16), *LAMA5* (18.75%, 3/16), *NBPF10* (18.75%, 3/16), *AHNAK* (18.75%, 3/16), *ZNF43* (18.75%, 3/16), *MUC12* (18.75%, 3/16), *ZNF737* (18.75%, 3/16), *ZNF208* (18.75%, 3/16), and *FLG2* (18.75%, 3/16).

**Table 1:** The clinical characteristics of 16 high-grade serous carcinoma patients

ID	Group	Age (year)	Stage	Residual lesion	Sreum CA125(IU/ml)	PFS (months)	OS (months)
Pt01	R	69	IIIC	R1	1808	0	16
Pt02	R	60	IIIC	R0	946	4	S*
Pt03	R	49	IIIC	R1	368	4	15
Pt04	R	69	IIIC	R1	1396	3	S*
Pt05	R	62	IIIA	R1	984	5	28
Pt06	R	54	IIIC	R0	348	0	28
Pt07	R	57	IIIC	R0	1758	4	52
Pt08	S	41	IIIC	R1	328	13	S*
Pt09	S	65	IIIC	R1	2023	19	30
Pt10	S	53	IIIC	R1	1397	13	42
Pt11	S	63	IVA	R1	30	16	32
Pt12	S	42	IIIC	R1	216	13	S*
Pt13	S	66	IIIB	R1	3092	17	S*
Pt14	S	53	IIIC	R0	473	22	S*
Pt15	S	36	IVA	R1	2395	17	S*
Pt16	S	47	IIIC	R1	4322	14	36

*R* Platinum resistance group, *S* Platinum sensitive group, *R0* No macroscopic residual disease, *R1* Residuals 1-10 mm, S* survival

### Neoantigens and tumor mutational burden distribution in epithelial ovarian cancer patients

**Fig. 1:**
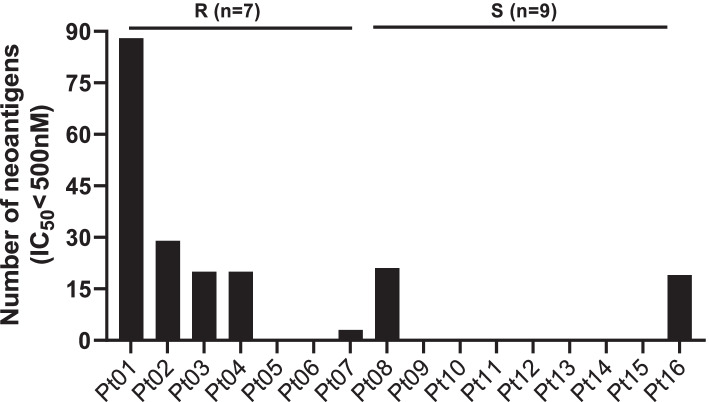
Number of neoantigens in the 16 patients. Note: Pt01~Pt16, patients ID. R, platinum-resistant group. S, platinum-sensitive group. IC50, half maximal inhibitory concentration, which represents the affinity between neoantigen peptide and MHC

Based on WES, RNA-seq and HLA typing, 7 of the 16 patients had predicted neoantigens, and the detection rate of neoantigens was 43.75% (HLA I, IC_50_<500 nM). The number of putative mutated neoantigens ranged from 3~88 (HLA I, IC_50_<500 nM). Among the 7 patients who had neoantigens detected, 5 were in the platinum-resistant group (range: 3-88), and 2 were in the platinum-sensitive group (19 and 21) (Fig. 1). The number of neoantigens in Group R was higher than that in Group S. However, there was no significant difference in the number of neoantigens between the two groups (*P*=0.1086) for IC_50_<500 nM (Table 2).

**Table 2:** TMB, neoantigen, immune score and ITH in Groups R and S

Variables	R (*n*=7)	S (*n*=9)	*P* value
Neoantigen			
Range	0~88	0~21	0.1086
Median	20	0	
TMB			
Range	0.14~1.86	0.05~1.62	0.0321
Median	1.43	0.34	
Immune score			
Range	-829.33~1398.51	-362.67~1783.08	0.7221
Median	196.60	301.05	
ITH			
Range	35.94~95.56	0~89.88	0.0452
Median	61.78	37.85	

*TMB* tumor mutational burden, *ITH* intratumor heterogeneity

**Fig. 2:**
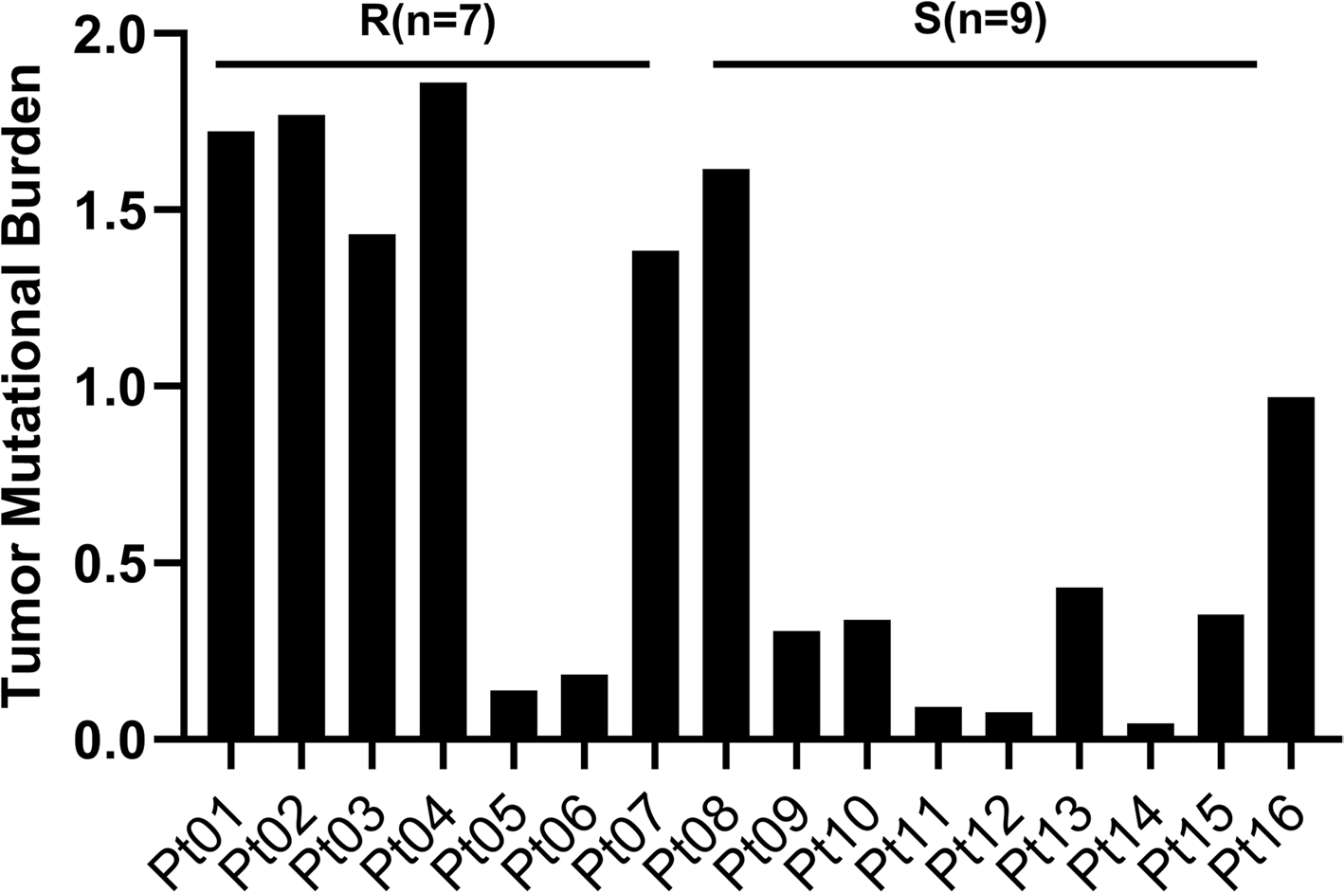
Tumor mutational burden (TMB) in the 16 patients. Note:Pt01~Pt16, patients ID. R, platinum-resistant. S, platinum-sensitive

Tumor mutational burden (TMB) was also measure for each patient. The TMB value in Group R ranged from 0.14 to 1.86 with a median of 1.43, and that in Group S ranged from 0.05 to 1.62 with a median of 0.34 (Fig. 2). There was a significant difference between the two groups (*P*=0.0321) (Table 2).

The ‘immune score’ of the two groups was evaluated by ESTIMATE software. The median ‘immune score’ in Group R was 196.6 while that in Group S was 301.05, no significant difference was observed (*P*=0.7221) (Table 2).

In addition, based on the MATH algorithm, the ITH of the two groups was evaluated. The median ITH of patients in Group R was 61.78, and that of Group S was 37.85, with a significant difference between the two groups (*P*=0.0452) (Table 2).

**Fig. 3:**
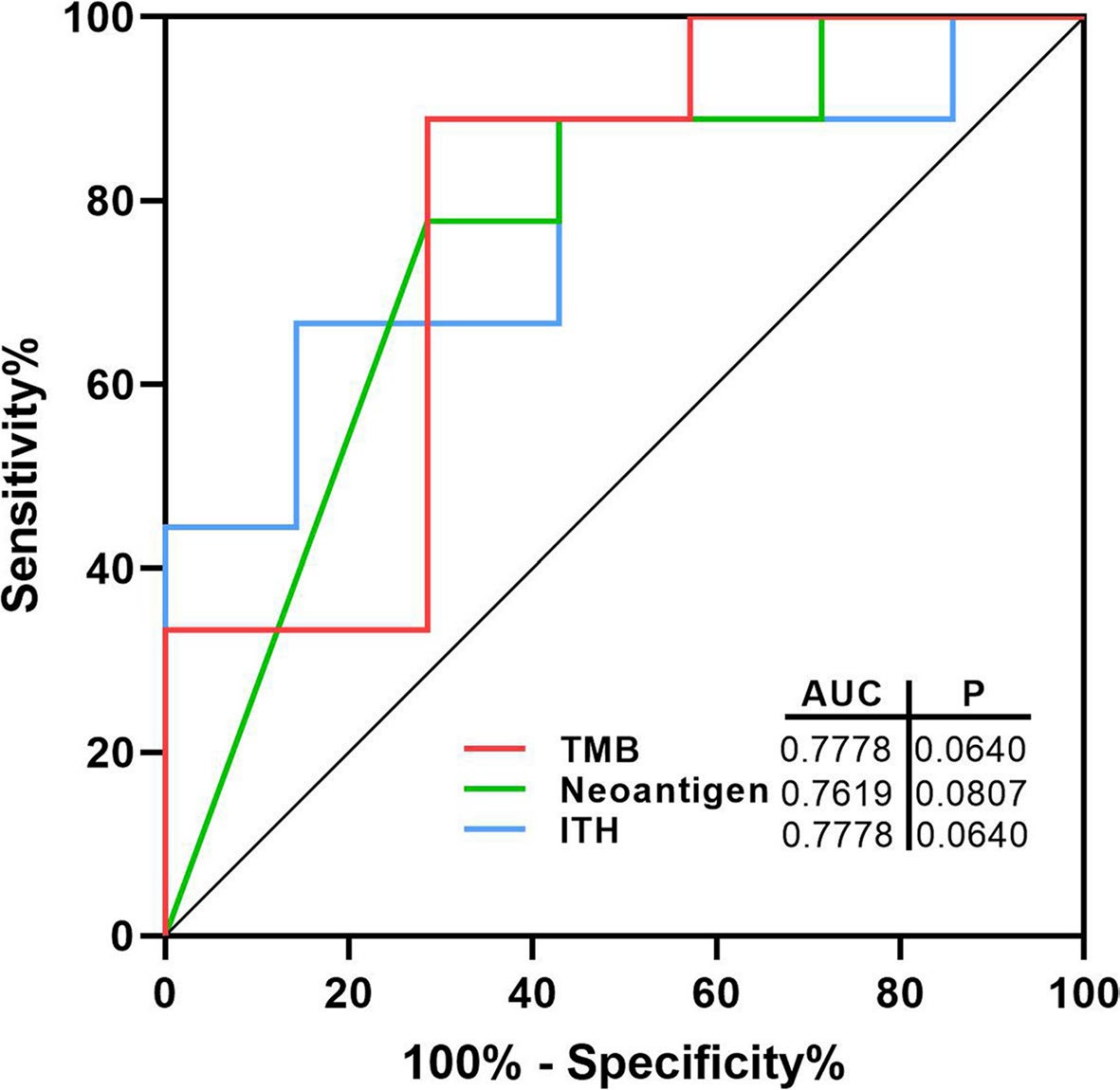
Receiver operating characteristic (ROC) curves of TMB, neoantigen, ITH and the area under the curve (AUC) for TMB, neoantigen and ITH. Red line, the ROC curve of TMB. Green line, the ROC curve of neoantigen. Blue line, the ROC curve of ITH

To determine the optimal cutoff points for predictive biomarkers and sensitivity to platinum, the predictive abilities were evaluated for TMB, neoantigen and ITH with using receiver operating characteristic (ROC) curves. As shown in Fig. 3, the optimal cutoff points of TMB, neoantigen and ITH were approximately 1.18, 1.50 and 44.41, respectively (*P*=0.064, *P*=0.081, *P*=0.064). Moreover, the three biomarkers had certain predictive value according to the ROC curve (AUC: 0.7778 for TMB, 0.7619 for neoantigen, 0.7778 for ITH).

With the same screening criteria in this study, 13 patients with PFS <6 months (platinum-resistant group) and 80 patients with PFS >12 months (platinum-sensitive group) from the TCGA database were included for the analysis. TMB comparison between the two groups showed that the average TMB of the platinum-resistant group and sensitive group was 4.254 and 3.198, respectively, but the difference was not statistically significant (*P*=0.317) (Supplementary Fig. S2).

### Immune microenvironment and immunotherapy

Based on the MCP-counter software analysis, the distribution of immune cells in tumor tissues was calculated, including T cells, CD8+ T cells, cytotoxic lymphocyte, natural killer (NK) cells, B cells, monocytes, myeloid dendritic cells, neutrophils, endothelial cells and fibroblasts. The mean value of CD8+ T cells in Group S was 1.0, and that in Group R was 0.5. The mean value of T cells in Group S was 3.49, while that in Group R was 4.3, but there was no significant difference between the two groups. Furthermore, the infiltration of other immune cells in tumor tissues was different between the two groups, although this was not significant (Table 3).

**Table 3:** The infiltration of immune cells in tumor tissues in Groups R and S

Variables	R (*n*=7)	S (*n*=9)	*P* value
T cells			
Range	0.49~7.35	2.14~8.31	0.7661
Mean	4.30	3.94	
CD8+T cells			
Range	0~2.94	0~1.54	0.3286
Mean	0.5	1.0	
Cytotoxic lymphocytes			
Range	0.31~8.43	0.13~9.69	0.9675
Mean	3.47	3.41	
NK cells			
Range	0~2.29	0.55~1.59	0.5747
Mean	0.98	1.19	
B cells			
Range	5.85~303.83	2.33~1254.53	0.2714
Mean	275.84	94.9	
Monocytes			
Range	4.19~20.96	4.47~24.65	0.4863
Mean	12.74	10.52	
Myeloid dendritic cells			
Range	0.07~7.18	0.56~6.17	0.8050
Mean	2.77	2.53	
Neutrophils			
Range	3.5~8.06	3.29~15.90	0.8522
Mean	6.09	5.79	
Endothelial cells			
Range	3.63~15.73	4.90~11.80	0.3750
Mean	7.20	8.88	
Fibroblasts			
Range	75.32~854.11	178.84~618.35	0.5737
Mean	357.56	482.73	

Nevertheless, further analysis was carried out to explore the correlation of clinical variables with different PFS. Univariate Cox regression analysis was performed with the immunotherapy-related variables, and the results showed significant associations between PFS and the number of neoantigens (*P*=0.0069), between PFS and TMB (*P* =0.0073), and between PFS and ITH (*P*=0.0249), whereas age, serum CA125 value and each kind of immune cell were not significantly related to PFS (Table 4).

**Table 4:** Univariate cox regression analysis for risk factors associated with PFS

Variables	HR	CI (95%)	*P* value
Age (year) Serum CA125 (IU/ml) Neoantigen (<500nM)	1.010 1.000 1.050	0.960-1.060 0.999-1.000 1.010-1.080	0.6908 0.6151 0.0069
TMB	4.060	1.460-11.300	0.0073
Immune score	1.000	0.999-1.000	0.5977
ITH	1.020	1.000-1.050	0.0249
T cells	1.010	0.798-1.280	0.9241
CD8+ T cells	0.795	0.462-1.370	0.4074
Cytotoxic lymphocytes	1.090	0.894-1.330	0.3938
NK cells	0.556	0.273-1.130	0.1061
B cells	1.000	0.999-1.000	0.3662
Monocytes	1.020	0.922-1.130	0.6778
Myeloid dendritic cells	1.020	0.723-1.430	0.9185
Neutrophils	1.060	0.854-1.320	0.5943
Endothelial cells	0.931	0.807-1.070	0.3251
Fibroblasts	0.999	0.997-1.000	0.2074

*TMB* tumor mutational burden, *ITH* intratumor heterogeneity

### Differentially expressed gene analysis and functional enrichment analysis

Differentially expressed genes (DEGs) and functional enrichment of the platinum-resistant group and platinum-sensitive group were analyzed in this study. The results illustrated that 200 genes were upregulated, and 954 genes were downregulated in platinum-resistant tumor samples compared with platinum-sensitive tumor tissues.

**Fig. 4:**
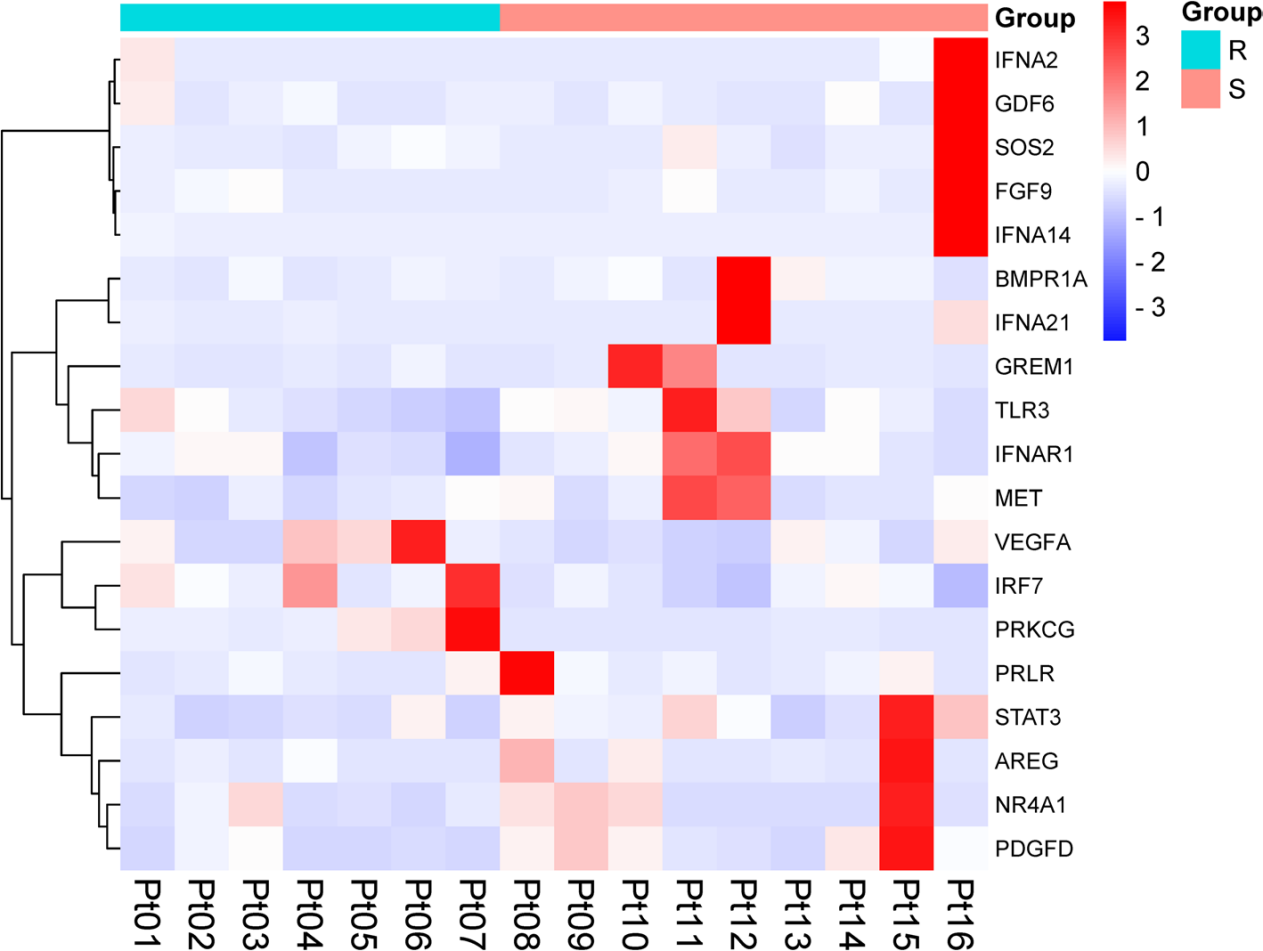
Nineteen immune-related genes between the R and S groups. R, platinum-resistant group. S, platinum-sensitive group. Note: blue—low expression, red—high expression. All 19 immune-related genes were significantly enriched in the PI3K-Akt signaling pathway, focal adhesion pathway, HIF-1 signaling pathway, MAPK signaling pathway, Toll-like receptor signaling pathway, and TGF-beta signaling pathway

**Fig. 5:**
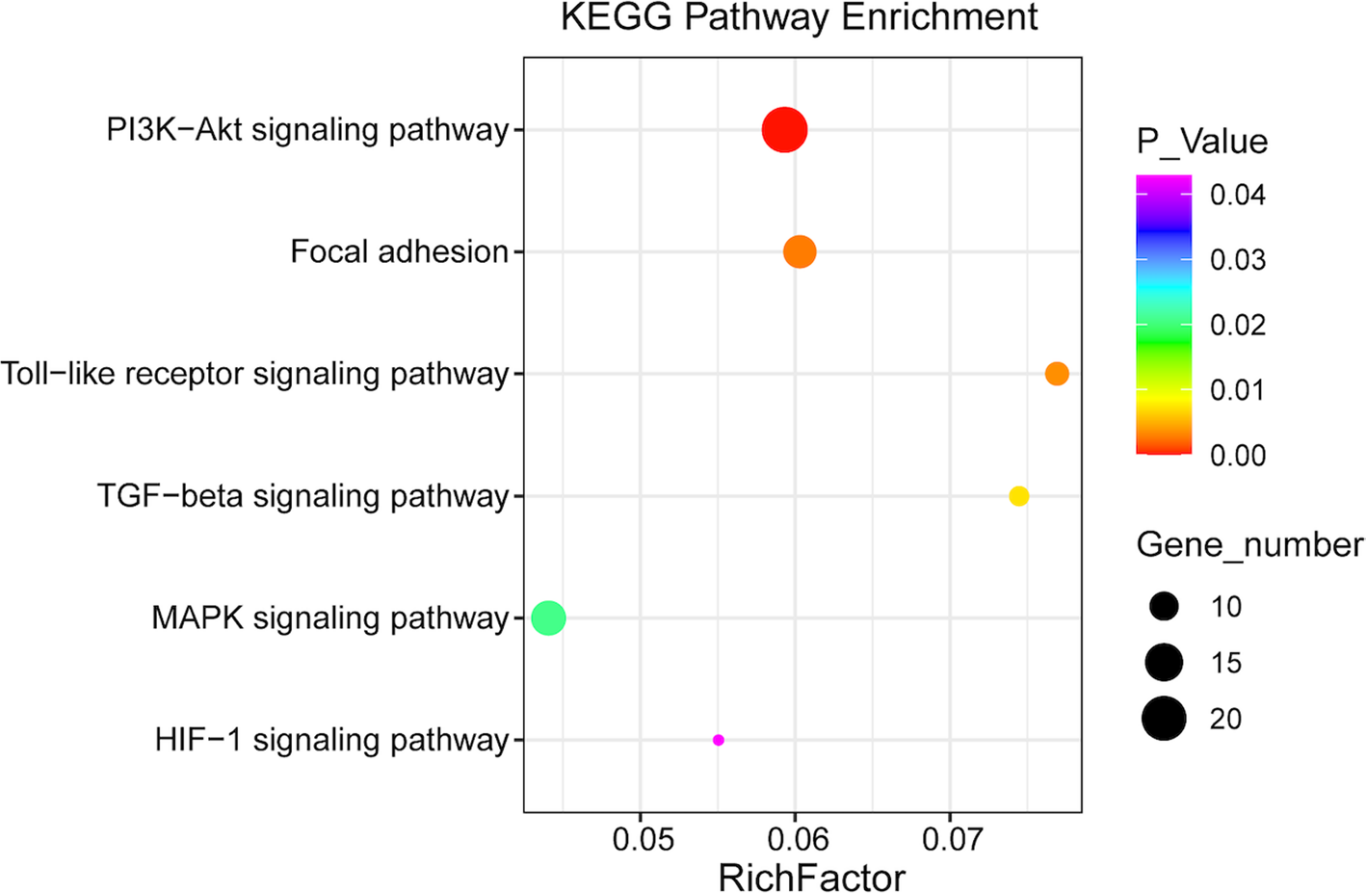
Bubble chart of six enriched Kyoto Encyclopedia of Genes and Genomes (KEGG) pathways reported to be associated with chemo resistance

Then, Kyoto Encyclopedia of Genes and Genomes (KEGG) analysis was performed to explore altered pathways, and 85 pathways were found to be significantly enriched. Of these pathways, six were associated with chemoresistance, including the phosphatidylinositol 3-kinase-Akt (PI3K-Akt) signaling pathway, focal adhesion, hypoxia-inducible factor 1(HIF-1) signaling pathway, mitogen-activated protein kinase (MAPK) signaling pathway, Toll-like receptor signaling pathway, and transforming growth factor-beta (TGF-beta) signaling pathway. Base on the immune-related gene database, ImmPort (https://www.immport.org/), 19 immune-related genes enriched in those pathways were differentially expressed (Fig. 4). These 19 immune-related genes are categorized into cytokines (*AREG*, *FGF9*, *GDF6*, *GREM1*, *PDGFD*), antigen processing and presentation (*IFNA14*, *IFNA2*, *IFNA21*), cytokine receptors (*BMPR1A*, *MET*, *NR4A1*, *PRLR*), antimicrobials (*IFNAR1*, *IRF7*, *STAT3*, *TLR3*, *VEGFA*) and natural killer cell cytotoxicity (*PRKCG*, *SOS2*). The KEGG enrichment bubble plot is shown in Fig. 5.

## Discussion

The results of this study showed that TMB, neoantigen and ITH are significantly correlated with PFS in platinum-sensitive and platinum-resistant groups. Previous studies have shown that TMB is correlated with tumor behavior, for example, lung adenocarcinoma patients with higher mutational burden have a 14-month survival decrease [12], suggesting that high mutational burden may be a sign of poor prognosis. Similar to the results in this study, high-grade serous ovarian cancer patients in the platinum-resistant group with the same stage and treatments may have poor prognosis because of higher TMB. Moreover, the ITH is another metric to measure tumor malignancy, and it has been found that high levels of ITH are associated with poor prognosis. Yochai Wolf suggested that diminishing tumor heterogeneity limits the response of tumor cells to immunotherapy by reducing their neoantigen landscape; thus increasing reactive neoantigens may expose tumor cells to immune detection [13]. Our results are consistent with a previous study showing that all patients who develop platinum resistance have higher levels of ITH with short PFS. Although the P values of the three biomarkers were not significantly different, this may be attributed to the small number of cases, as the area under the ROC curve all met all the conditions. Thus, HGSC patients with TMB>1.18, ITH>44.41 and neoantigen>1.50 may be predicted to have a poor prognosis.

Moreover, studies have shown that platinum resistant mechanisms are related to many pathways, and the similar results were observed in this study. The results of this study show that DEGs between the resistant and sensitive groups are enriched in the PI3K-Akt signaling and epidermal growth factor receptor (EGFR) pathways. Recently, Yoshihara reported that ovarian cancer-associated platinum resistance in peritoneal metastasis was related to PI3K-Akt signaling. Additionally, in Liu XY’s study, platinum-resistance was observed to be related to PI3K-Akt signaling and focal adhesion pathways in triple-negative breast cancer patients [14, 15]. Other studies have found that platinum-resistance is related to the HIF-1 signaling pathway, MAPK signaling pathway, TGF-beta signaling pathway and Toll-like receptor signaling pathway [16–19]. Our study also suggests that DEGs of platinum resistance in ovarian cancer are enriched in these pathways. The mechanism of platinum resistance in ovarian cancer needs to be further clarified, which could provide a foundation for future immunotherapy research.

Neoantigens derived from tumor specific mutations are good potential targets for effective antitumor immune responses as they are foreign to the immune system [20]. Previous studies have suggested that patients with higher TMB, such as melanoma or smoking-related lung cancer, have higher rates of response to immune checkpoint blockade therapy, which is perhaps due to increased neoantigen expression [21–24]. Nevertheless, the results in a phase II trial for ovarian cancer shows that the response rate to pembrolizumab for HGSC was only 8% [25]. We compared the TMB and potential neoantigens in the two groups to determine which patients would be the best candidates for immunotherapy, and the results showed that patients in the platinum-resistant group had more potential neoantigens and had a higher TMB than those in the platinum-sensitive group. The response rate to immunotherapy for ovarian cancer is lower than that for other tumors, but the results of this study suggest that TMB can also be a promising biomarker for ovarian cancer patients to predict the prognosis and immunotherapy.

Although few ovarian cancer patients benefit from immunotherapy, we still try to identify people who could benefit from it. As reported previously, tumor mutational burden (TMB) correlates with tumor behavior and immunological response [23, 26, 27]. The relationship between TMB and neoantigens is a research hotspot. TMB can indirectly reflect the ability and extent of neoantigens produced by tumors and has been used to predict immunotherapeutic response in a variety of cancers. One study indicated that highly mutated tumors may develop many novel peptides and thus display more neoantigens, making them targets of activated immune cells [21, 23, 28]. For instance, patients with melanomas who have a high mutational load were observed to have improved survival with ipilimumab therapy and improved overall survival [29]. Indeed, higher TMB and neoantigen load have been associated with an enhanced response to immune checkpoint blockade therapy. The results of this study also show that patients with shorter PFS have more neoantigens and higher TMB, for whom early immunotherapy with chemotherapy might have a better treatment effect. Furthermore, the comparison of the TMB of HGSC patients in the TCGA database was consistent with this study. The TMB value of the platinum-resistant group was higher than that of the platinum-sensitive group, but there was no significant difference. This may be because the number of platinum-resistant groups and platinum-sensitive groups in the TCGA database is quite different, but their ratio in this study was essentially equal. Moreover, the Asian population selected from this study may have been different from the TCGA database. Therefore, the sample size of this study should be expanded for further research.

In addition, to reveal suitable immunotherapy, immune cells infiltration in the tumor tissue was examined. Previous studies have suggested that immunotherapies that improve patients’ antitumor immune responses can result in significantly improved treatment outcomes [30–33]. When host antitumor immune function is inhibited, adoptive T cell therapy with neoantigen-specific T cell receptor (TCR)-engineered T cell can kill tumor cells. In Sato’s study, epithelial ovarian carcinoma patients with increased intraepithelial CD3+ and/or CD8+ tumor infiltrating lymphocytes (TILs) had better clinical treatment effects [34]. In this study, patients in the platinum-sensitive group had more CD8+T cell infiltration than those in the platinum-resistant group. Interestingly, patients in the platinum-resistant group had more T cells than patients in the platinum-sensitive group. It has been reported that neoantigens derived from somatic mutations are well-recognized as good targets for T cells [23, 35, 36]. In that case, immunotherapy might be applied to patients in the platinum-resistant group as early as possible to promote the CD8+/T cell ratio in order to improve the treatment effect.

This was a retrospective study. The high cost of sequencing technology and a certain failure rate for DNA and RNA extraction and library construction both led to a small number of patients enrolled in this study. The sample size needs to be expanded, and more research is necessary to clarify the immunotherapeutic mechanisms of neoantigens in the future.

## Conclusion

Higher TMB, neoantigens and ITH may account for the worse prognosis of HGSC patients treated with platinum-based chemotherapy. Since higher TMB is a biomarker for successful immunotherapy and higher TMB was observed in the platinum-resistant group, patients in the platinum-resistant group could be the better candidates for immunotherapy. Neoantigen and T cell infiltration might enhance the immunotherapy effect in the platinum-resistant group.

## Supplementary Information

**Additional file 1: Supplementary Figure S1.** The mutational landscape of 16 patients. The top 14 mutated genes (mutated sample number ≥3) in these 16 patients are shown.

**Additional file 2: Supplementary Figure S2.** TMB comparison of HSGC patients with PFS < 6 months and PFS > 12 months in The Cancer Genome Alas (TCGA) database

## Data Availability

The data that support the findings of this study are not publicly available due to their containing information that could compromise the privacy of research participants but are available on request from the corresponding author.
