# Supplementary figures and images for: Neoantigens and the tumor microenvironment play important roles in the prognosis of high-grade serous ovarian cancer
Source: J Ovarian Res. 2022 Jan 29;15:18. doi: 10.1186/s13048-022-00955-9 (PMC8801100; doi:10.1186/s13048-022-00955-9)

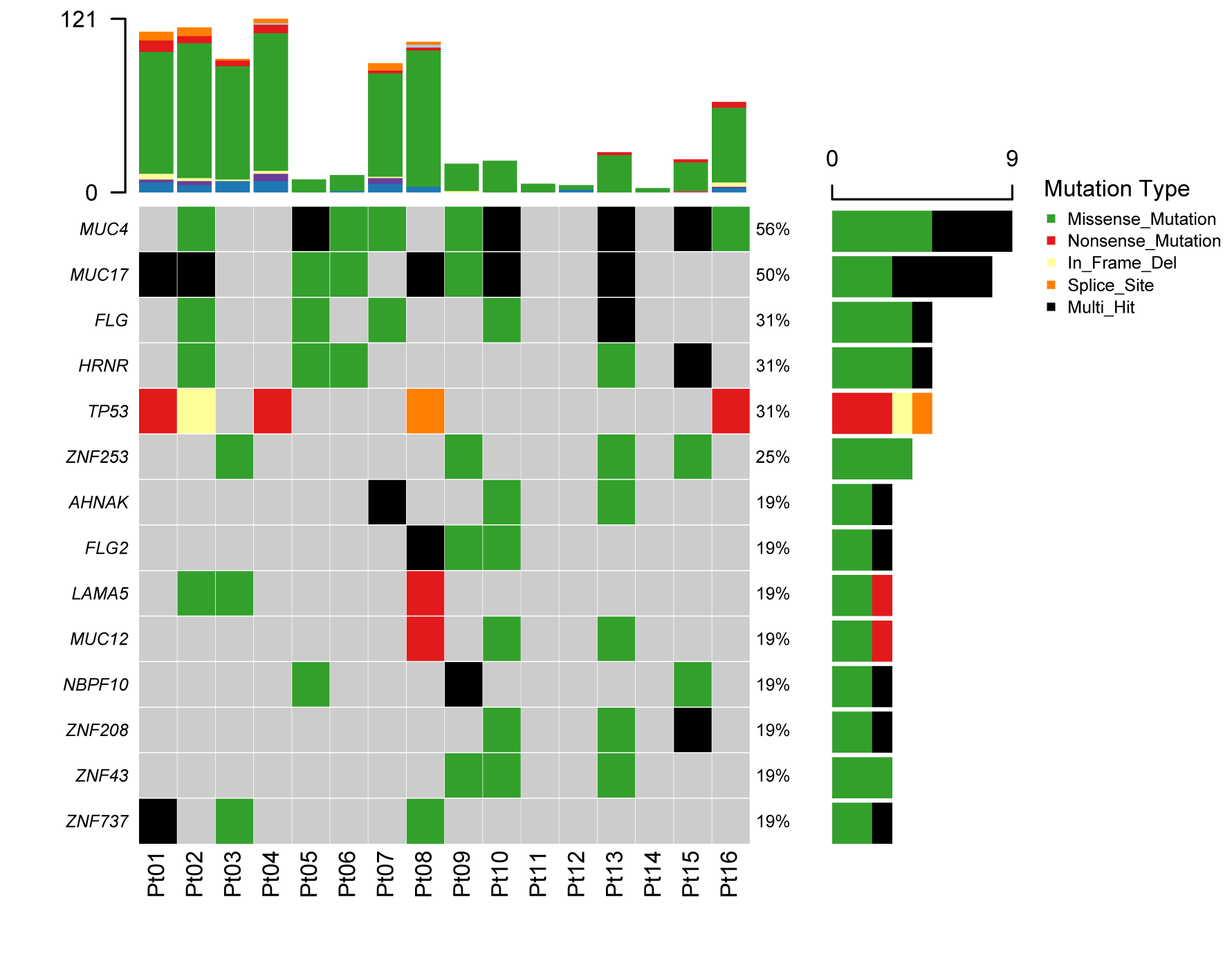

Supplement: Supplementary file 1 — Additional file 1: Supplementary Figure S1. The mutational landscape of 16 patients. The top 14 mutated genes (mutated sample number ≥3) in these 16 patients are shown. [file 13048_2022_955_MOESM1_ESM.tif]

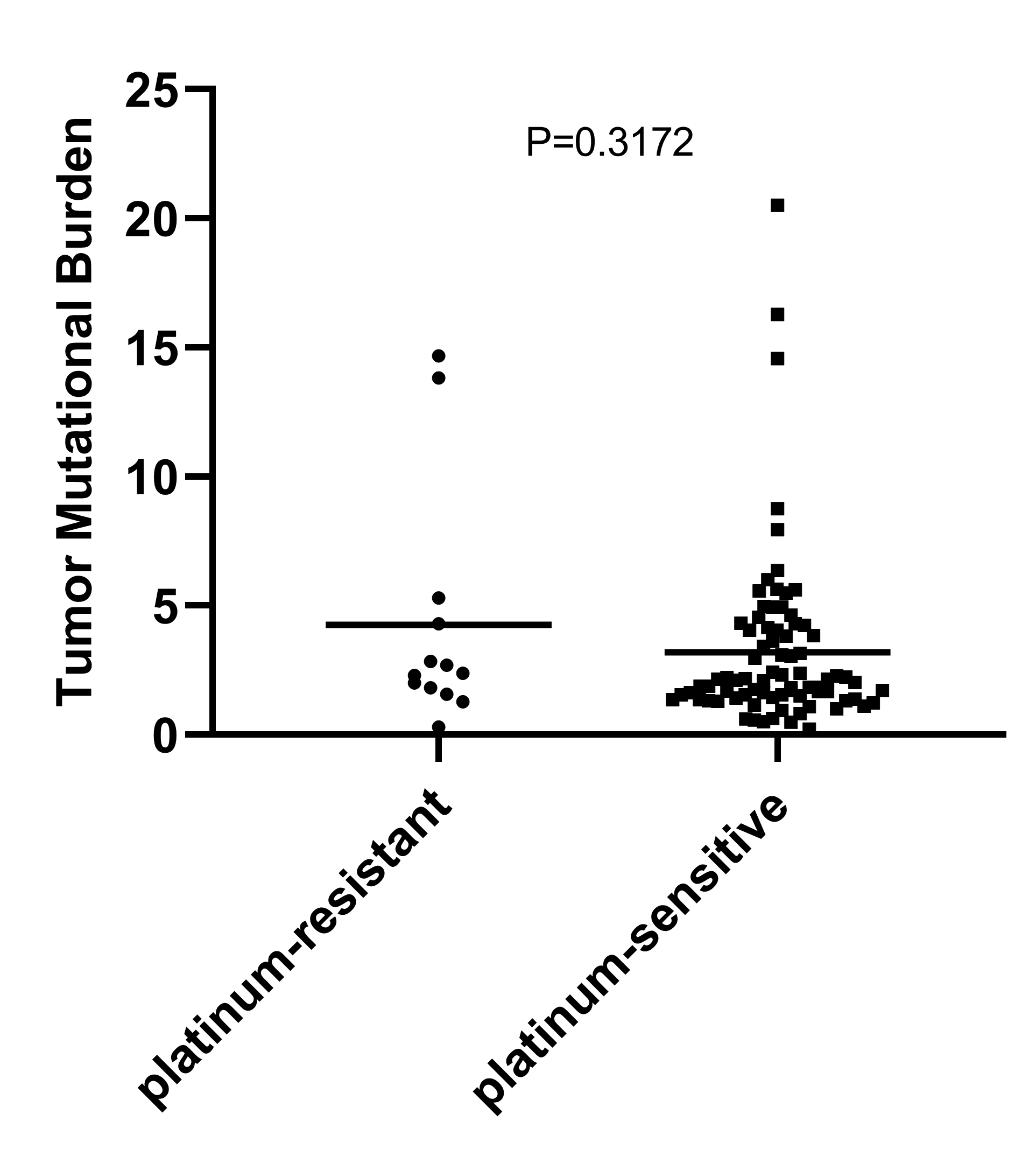

Supplement: Supplementary file 2 — Additional file 2: Supplementary Figure S2. TMB comparison of HSGC patients with PFS < 6 months and PFS > 12 months in The Cancer Genome Alas (TCGA) database [file 13048_2022_955_MOESM2_ESM.tif]
